# Improvement in symptoms of anxiety and depression in individuals with type 2 diabetes: retrospective analysis of an intensive lifestyle modification program

**DOI:** 10.1186/s12888-024-06130-2

**Published:** 2024-10-22

**Authors:** Pramod Tripathi, Baby Sharma, Nidhi Kadam, Diptika Tiwari, Thejas Kathrikolly, Anagha Vyawahare, Mayurika Das Biswas, Venugopal Vijayakumar, Maheshkumar Kuppusamy, Malhar Ganla, Banshi Saboo

**Affiliations:** 1 Department of Research, Freedom from Diabetes Research Foundation, Pune, Maharashtra 411004 India; 2 Freedom from Diabetes Clinic, Pune, Maharashtra India; 3 Department of Yoga, Government Yoga and Naturopathy Medical College and Hospital, Chennai, Tamil Nadu India; 4 Department of Physiology, Government Yoga and Naturopathy Medical College and Hospital, Arumbakkam, Chennai, Tamil Nadu India; 5 Department of Medicine, Dia Care- Diabetes Care and Hormone Clinic, Diabetology, Ahmedabad, Gujarat India

**Keywords:** Anxiety, Depression, Type 2 diabetes, Mental health, Intensive lifestyle intervention, India

## Abstract

**Background:**

Type 2 diabetes (T2D) is a chronic metabolic disorder that has a notable influence on mental well-being, contributing to elevated morbidity and mortality rates. Depression and anxiety disorders are the most common mental health concerns among patients with T2D worldwide. Therefore, the present study aimed to assess the impact of an online intensive lifestyle intervention (ILI) on mental health outcomes (depression and anxiety) in patients with T2D in India.

**Materials and methods:**

This retrospective pre-post analysis included adult patients (aged > 18 years) diagnosed with T2D who were enrolled in a diabetes management program in India between June 2021 and June 2023. The intervention consisted of lifestyle modifications such as a customized plant-based diet, regular physical activity, psychological support through group and individual therapy, and medical management. Data were extracted from the electronic database of the clinic, including anthropometry, medical history, biochemical parameters, symptoms of depression, and anxiety (assessed using the Patient Health Questionnaire-9 (PHQ-9) and Generalized Anxiety Disorders-7 (GAD-7) scale) at the start and end of the six-month intervention period.

**Results:**

Of the 1061 eligible patients (177 with prediabetes), 40.3% were female. The mean age, duration of diabetes, and HbA1c levels were 52 ± 10.4 years, 9.8 ± 7.8 years, and 8 ± 1.7%, respectively. The prevalence of symptoms of depression and anxiety (ranging from mild to severe) was 46% and 44.3%, respectively, which reduced to 28.7% and 29.2%, respectively, post-intervention.

**Conclusion:**

Integrated ILI successfully improved the symptoms of anxiety and depression, highlighting the importance of a multidisciplinary approach that includes diet, physical activity, psychological support, and medical management in enhancing mental health outcomes among patients with T2D. Future prospective studies are needed to explore the long-term effects of such interventions and develop effective strategies for promoting mental health in diverse populations.

**Trial registration:**

The study was approved by the Freedom from Diabetes Research Foundation Institutional Ethics Committee (approval number FFDRF/IEC/2024/7) and registered in the Clinical Trials Registry of India (CTRI/2024/03/064596, registered on March 21, 2024).

## Background

Mental well-being is crucial for managing various health conditions, including Type 2 diabetes (T2D) [1, 2]. T2D is a chronic metabolic disorder that affects millions of people worldwide and is associated with increased morbidity and mortality [3]. It affects not only the physical health of individuals but also their mental health, as patients often experience psychological distress, anxiety, and depression [4]. These mental health issues can impair the quality of life, self-care behaviors, and glycemic control of patients [5].

Depression and anxiety disorders are the most prevalent forms of psychological distress among T2D patients globally [6]. Further, there is a documented reciprocal relationship between T2D and depression, indicating that diabetes can contribute to depression and vice versa [7, 8]. According to the American Diabetes Association (ADA), psychological conditions can appear in people with diabetes of all ages [4]; therefore, a comprehensive medical evaluation of patients with diabetes should include an assessment of psychosocial/emotional health concerns if indicated [9, 10].

A systematic review indicated that, on a global scale, approximately 28% of individuals with T2D experience depression and 14% have anxiety disorders associated with hyperglycemia [11]. In patients with diabetes, anxiety and depression have been linked to higher medical care expenses, interference with daily life routines, poorer quality of life [12], and an increased risk of complications [13]. Effective diabetes management relies on self-care and adherence to prescribed treatment to avoid complications. Psychological distress, particularly depression and anxiety, hinders self-care routines and compliance with treatment protocols [14, 15], which may worsen the prognosis of diabetes [16]. Therefore, the importance of addressing psychological distress in individuals with diabetes cannot be overstated, as it not only affects their ability to manage their condition but can also have negative consequences on their overall health and well-being. Considering the high burden of mental health conditions in patients with T2D, there is an urgent need to implement effective interventions and treatments that can improve their mental well-being.

Several interventions have been proposed to address the psychological needs of T2D patients, such as pharmacological treatment, cognitive behavioral therapy, collaborative care, and health education [17–20]. However, these interventions may have limitations, such as side effects, cost, accessibility, and adherence [21]. Additionally, there is a scarcity of research examining the effectiveness of a multidisciplinary approach for managing mental health issues in patients with T2D, especially considering the complex and reciprocal relationship between T2D and mental health outcomes. Lifestyle changes such as modifications to diet and exercise are crucial for managing T2D, but their comprehensive impact on both T2D management and mental health remains underexplored [22]. Therefore, this study aimed to retrospectively assess the impact of an integrated intensive lifestyle intervention (ILI) (including diet, exercise, psychological support, and medical support) in alleviating symptoms of anxiety and depression in individuals with T2D.

## Methods

### Study design and setting

This retrospective study was carried out at the Freedom from Diabetes Clinic, which operates on a one-year subscription-based program for the online management of diabetes. The eligibility criteria included participants aged > 18 years with a confirmed diagnosis of T2D (all patients on treatment with oral hypoglycemic agents and/or Insulin or with HbA1c ≥ 6.5% without medication) or prediabetes (HbA1c between 5.7 and 6.5%) [23]. Patients with a history of medically diagnosed psychiatric illness (verified with medical records) and pregnant and lactating women were excluded.

**Fig. 1:**
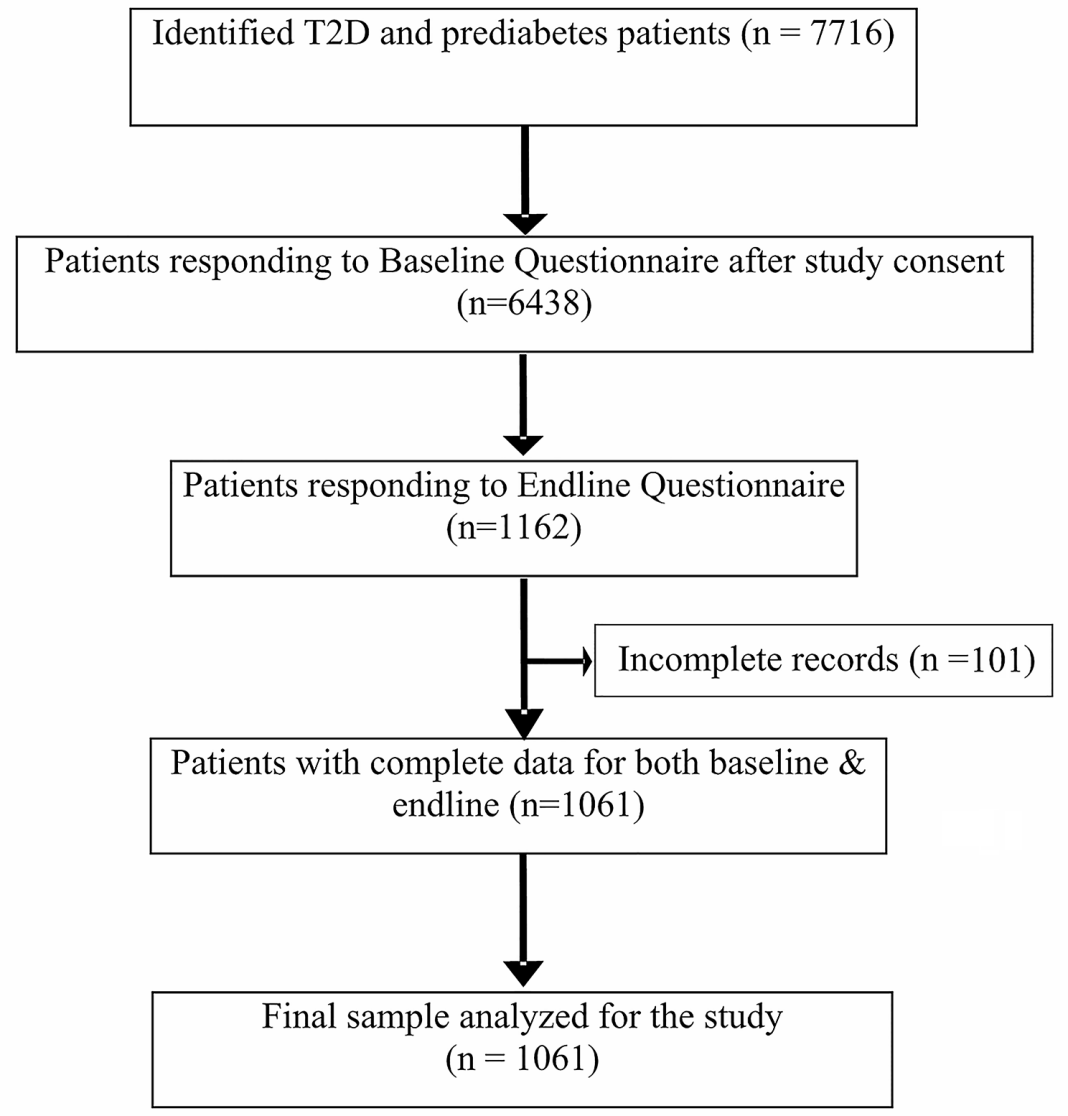
Flowchart depicting the selection procedure of patients for the study

Medical records of individuals with T2D enrolled between June 2021 and June 2023 (*n* = 7716) were extracted from the electronic database of the Freedom from Diabetes Clinic in Pune, India. All patients (*n* = 1061 across 175 cities in India) with complete medical records, who met the eligibility criteria and had pre-post data on depression and anxiety scales, were included in the final analysis. The patient flow chart for this study is shown in Fig. 1.

### Ethical consideration

This retrospective study adhered to the ethical principles outlined in the Declaration of Helsinki. This study was approved by the Freedom from Diabetes Research Foundation Institutional Ethics Committee (approval number: FFDRF/IEC/2024/7). This study was registered with the Clinical Trials Registry of India (CTRI) under registration number CTRI/2024/03/064596 on March 21, 2024. Owing to the retrospective design of the study, the requirement for informed consent from participants was waived by the ethics committee.

### Tools used for data collection

The de-identified data of 1061 participants were retrospectively extracted from electronic records maintained by the clinic. Collected data included sociodemographic (sex, age, marital status, education level, occupation, and family history), anthropometric (height and weight), medical history (date of diabetes detection, associated comorbidities, and medication status), and biochemical parameters (HbA1c, Fasting Blood Glucose (FBG), and postprandial blood glucose (PPBG)).

Additionally, the scores of the Patient Health Questionnaire-9 (PHQ-9) and Generalized Anxiety Disorder-7 (GAD-7) scales were included to assess depression and anxiety levels, respectively (data on the two scales were routinely collected as part of the program). Depression severity was assessed using the Patient Health Questionnaire (PHQ-9), a 9-item self-report screening tool. The PHQ-9 uses a 4-point scale ranging from 0 to 3, where 0 signifies “never,” 1 signifies “several days,” 2 signifies “more than half the time,” and 3 signifies “nearly every day.” The total score for the PHQ-9 ranges from 0 to 27, with higher scores indicating greater severity of depression symptoms. The PHQ-9 has established cutoff scores of 5 for mild depression, 10 for moderate depression, 15 for moderately severe depression, and 20 for severe depression. The PHQ-9 has demonstrated a high level of sensitivity (88%) and specificity (88%) for detecting depression [24].

The Generalized Anxiety Disorder (GAD-7), a 7-item self-report screening tool, was used to evaluate anxiety symptoms. Each item on the GAD-7 scale was rated on a 4-point scale from 0 to 3, with 0 representing “never,” 1 representing “several days,” 2 representing “more than half the time,” and 3 representing “nearly every day.” The overall score ranged from 0 to 21, with higher scores indicating greater symptom severity. Cut-offs of 5, 10, and 15 represented mild, moderate, and severe anxiety, respectively. The GAD-7 has a sensitivity of 89% and specificity of 82% for detecting anxiety [25].

### Study intervention

The detailed intervention has been previously described [26]. The intervention comprised of four main components: diet, physical activity, psychological support, and medical management. Upon enrollment, each patient was assigned a team of six specialists: physician, dietician, physiotherapist (exercise expert), psychologist, mentor (former program participant who volunteered to guide new patients), and monitor (for follow-ups and reminders for lab tests, appointments, etc.). The intervention was delivered through online group sessions (educational) and individual consultations with each of the four primary experts.

The implemented dietary modifications consisted of a plant-based diet [26]. The goal of this dietary intervention was to detoxify and alkalize [27, 28] the body while facilitating weight loss through a gradual decrease in calorie intake achieved with intermittent fasting [29]. Once the target weight was achieved, the focus shifted towards building muscle through a diet with increased protein intake. This dietary approach was complemented with exercise recommendations. During the initial weight-loss period, the exercises focused on building strength, stamina, and flexibility [26]. After reaching the target weight, the focus shifted to muscle building. Overall, both the diet and exercise plans were individualized to consider the age, sex, fitness level, and comorbidities of each participant.

The psychological intervention aimed to assess participants’ stress and anxiety levels while simultaneously fostering an understanding of the mind-body connection. Each month, a team of psychologists conducted group therapy sessions with different themes. These themes ranged from guided meditations and the introduction of journaling to foster positive energy [30–32] through activities such as self-reflection and goal-setting techniques [32–34] using vision boards [35] to support personal growth and well-being. Additionally, participants who scored high on the PHQ-9 and GAD-7 at baseline (scores above 10, indicating moderate to severe anxiety and/or depression) received individual counseling from their assigned psychologist. Specialized techniques tailored to each patient’s unique circumstances were employed. These techniques include Rational Emotive Behavior Therapy (REBT) to help participants identify and challenge irrational beliefs that contribute to emotional distress [36], Clinical Hypnotherapy to access the subconscious mind and address deep-seated psychological issues [37, 38], Neuro-Linguistic Programming (NLP) techniques to facilitate positive behavior changes through language and thought patterns [39], positive affirmations to help participants reframe negative self-talk, and meditation practices to enhance mindfulness and reduce stress [34]. Participants, especially those with a high depression score, were encouraged to keep gratitude journals to foster a positive outlook on life [32]. For those with high anxiety scores, Cognitive Behavior Therapy (CBT) was employed to identify and modify distorted thinking patterns [5, 18, 40], while breathwork techniques were used to promote relaxation and reduce stress [41]. Additionally, progressive muscle relaxation was introduced to reduce physical tension and anxiety [41]. Life coaching was provided to guide participants in achieving personal goals and overcoming obstacles [42], and *pranic healing* was included as a complementary approach to empower participants with overall well-being [43]. This customization ensured that the treatment aligned with each patient’s specific needs and comfort level.

Medical management involved daily blood glucose monitoring, addressing nutritional deficiencies through supplements, and at least 3 medical consultations with the assigned physician.

### Use of technology

The patients were given access to a dedicated ‘*Freedom from Diabetes’* mobile application (https://play.google.com/store/apps/details? id=com.ffd) that allowed them to communicate with the designated team of specialists via voice and video calls and text messages throughout the intervention period. The application was designed to facilitate regular monitoring and updating of vital signs, including FBG, PPBG, weight, and blood pressure, as well as diet and exercise. The physician used the app to closely monitor blood glucose levels and blood pressure, adjusting the daily medication doses as needed. Additionally, the application provided patients with access to plant-based recipes, recorded meditation sessions, and exercise videos.

Throughout the diabetes management program, patients received counseling and motivational support to encourage adherence to the protocol (via live online video conferencing, FFD mobile application, phone calls, and WhatsApp messages). The mobile application was used to conduct monthly adherence checks.

### Statistical analyses

In this retrospective analysis, all the data were thoroughly examined for completeness and accuracy. Following extraction in Microsoft Excel, the data were transferred to IBM SPSS (Statistical Package for the Social Sciences, for Windows, Version.21.0 IBM Corp., Armonk, New York, USA) for statistical analyses. Continuous variables are presented using descriptive statistics, with means and standard deviations for normally distributed variables (weight, BMI, HbA1c) and medians with interquartile ranges (IQRs) for skewed variables (anxiety and depression scores). Categorical variables are reported as frequencies and percentages. Classification of patients into mild to severe depression and/or anxiety groups is based on a cut-off score of 5 [24, 25]. To assess the effectiveness of the intervention in reducing depression and/or anxiety levels, pre-post analyses were conducted using paired t-tests for continuous variables and the McNemar test for categorical variables. R-Software was used for the violin plot to show the change in the score from the baseline to the endline. All analyses were two-tailed with a significance level of *p* < 0.05.

## Results

The mean age and diabetes duration of patients were 52 ± 10.4 years and 9.8 ± 7.8 years, respectively. The mean baseline weight (kg), BMI (kg/m^2^), and HbA1c (%) levels of the patients were 73.06 ± 14.5, 26.5 ± 4.5, and 8 ± 1.7, respectively. The majority of patients were male, married, salaried, had diabetes for more than five years, had a self-reported history of stress before diabetes, and had a family history of diabetes (Table 1). The majority (88.8%) of patients were on medication for diabetes (oral hypoglycemic agents (OHAs) and/or insulin), and most reported having one or more comorbidities (Table 1).

**Table 1:** Sociodemographic and biochemical characteristics of the study population

Parameter	Frequency (*N*)	Percentage (%)
**Age groups** ^a, b^		
Less than 45 years	257	24.2
45–55 years	377	35.5
55–65 years	320	30.2
More than 65 years	107	10.1
**Sex** ^**a, b**^		
Male	633	59.7
Female	428	40.3
**Marital Status** ^**b**^		
Unmarried	47	4.4
Divorced/Separated	22	2.1
Widowed	23	2.2
Married	969	91.3
**Education** ^**a, b**^		
Below Graduation	475	46.7
Above Graduation	542	53.3
**Occupation**		
Self Employed	228	21.5
Salaried	447	42.1
Retired	157	14.8
Other *	47	4.4
Homemaker	182	17.2
**Stress Before Diabetes (Self-reported)** ^**a, b**^		
No	343	32.3
Yes	718	67.7
**Family History**		
No	233	22
Yes	828	78
**BMI** [44]		
Normal (< 23 kg/m2)	232	21.9
Overweight (23–25 kg/m2)	210	19.8
Obese ( > = 25 kg/m2)	619	58.3
**Duration of diabetes** ^**a**^		
Less than 5 years	347	33.5
More than 5 years	689	66.5
**Glycemic Control** ^**b**^		
Good (HbA1c less than 7%)	350	33
Poor good (HbA1c more than 7%)	711	67
**Medication Usage** ^**a, b**^		
Insulin = Yes	149	14
Insulin = No	912	86
OHAs = Yes	943	88.9
OHAs = No	118	11.1
**Comorbidities** ^**a, b**^		
Hypertension = Yes	441	41.6
Hypertension = No	620	58.4
Dyslipidemia = Yes	644	60.7
Dyslipidemia = No	417	39.3

^a^Chi-square value significant for anxiety (*p* < 0.05), ^b^ Chi-square value significant for depression (*p* < 0.05); BMI: Body Mass Index; OHAs: Oral hypoglycemic agents; *Students/Do not wish to share

**Fig. 2:**
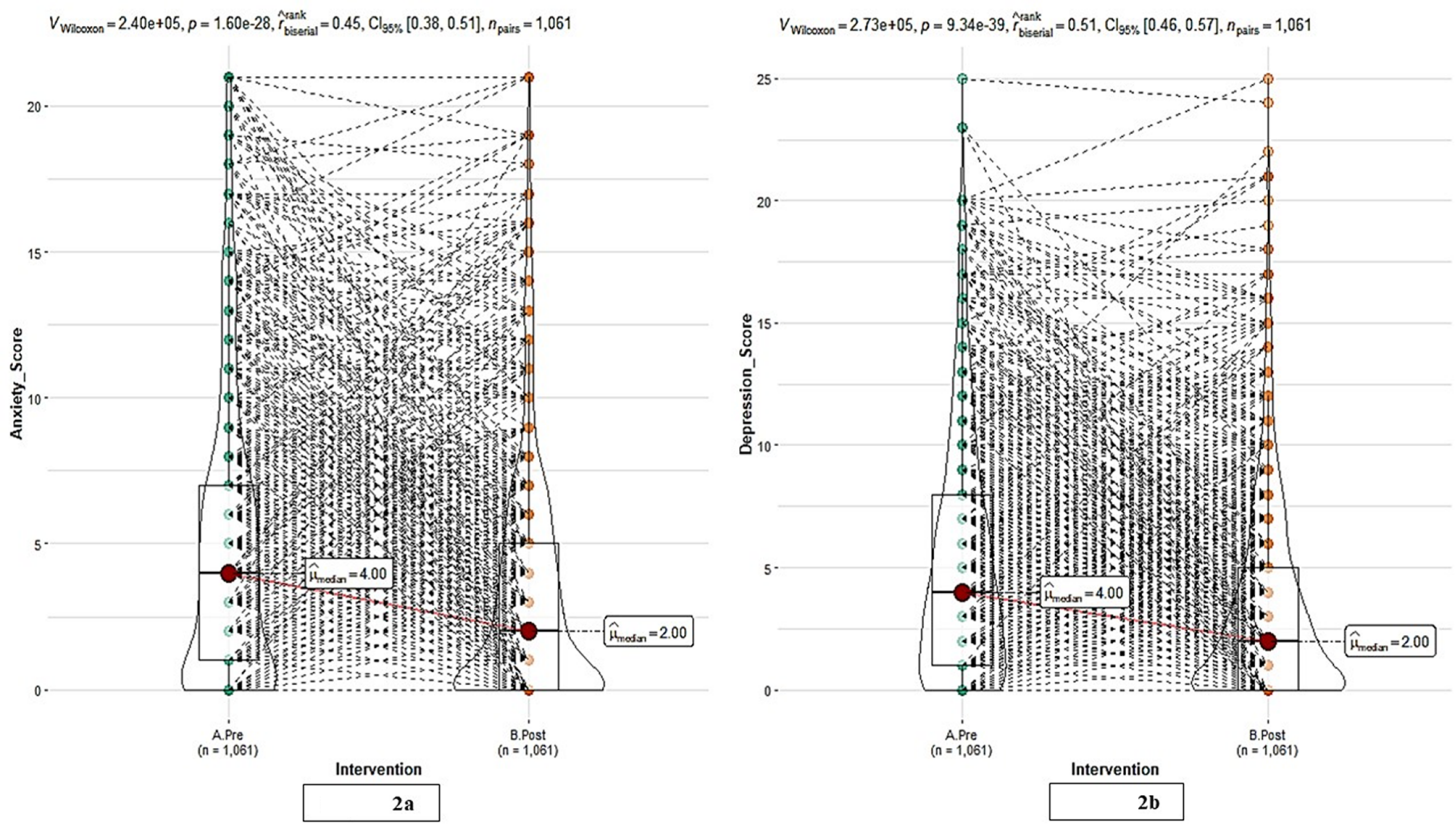
Post-intervention changes in scores in the overall cohort (**a**) anxiety scores (**b**) depression scores

Post-intervention, a statistically significant (*p* < 0.001) decrease in median weight was observed from 71 kg to 66 kg corresponding to an average reduction of 4.71 kg (95% CI: 4.3 to 5.0 kg). Similarly, median HbA1c levels showed a significant improvement (7.5 to 6.5%) (*p* < 0.001) following the intervention, with an average decrease of 1.28 (95% CI: 1.1 to 1.3). The effect sizes for the improvements in both weight and HbA1c levels were d = 0.8, indicating that the intervention had a strong and clinically meaningful impact on both weight reduction and improvement in HbA1c levels [45], with a large effect size underscoring the substantial impact of the intervention on these health parameters. Additionally, significant (*p* < 0.001) improvements in FBG (123 mg/dL to 114 mg/dL) and PPBG (150 mg/dL to 137 mg/dL) were observed. The effect size for the improvements in both FBG and PPBG were d = 0.3, indicating that while the intervention produced statistically significant improvements in both fasting and postprandial blood glucose levels, the magnitude of these improvements was relatively modest compared with the other outcomes measured. A significant reduction was observed in the overall score (Fig. 2) for both anxiety (Fig. 2a) and depression (Fig. 2b) (a decrease in the median score from four to two) in the entire cohort (*p* < 0.001). The effect size for this improvement was d = 0.3 for anxiety and d = 0.4 for depression, which indicates a small to moderate effect according to Cohen’s conventions [45]. This suggests that the intervention produced a modest but meaningful reduction in anxiety and depression symptoms in the overall cohort.

Furthermore, all patients were classified according to the severity of their anxiety and depression, ranging from mild to severe at baseline and endline (Tables 2 and 3). The prevalence of depression and anxiety symptoms (ranging from mild to severe) was 46% and 44.3% at baseline, which decreased to 28.7% and 29.2% post-intervention, respectively. The intervention notably improved the symptoms of severe anxiety and depression, with a substantial decrease in scores (8.79 and 9.08 points, respectively).

**Table 2:** Change in anxiety scores based on severity

Anxiety severity*	Pre-Intervention		Post-Intervention		Absolute Difference (95% CI)	*P* Value
	Median (IQR)	**N** (%)	Median (IQR)	**N** (%)		
Minimal (Score 0–4)	1 (0–3)	591 (55.7)	1 (0–3)	751 (70.8)	0.38 (-0.61 to 0.16)	0.05
Mild (Score 4–9)	6 (5–7)	302 (28.5)	4 (1–6)	224 (21.1)	2.34 (1.93 to 2.74)	< 0.001
Moderate (Score 10–14)	12 (11–14)	109 (10.3)	5 (2–7.5)	57 (5.4)	6.07 (5.14 to 7)	< 0.001
Severe (score 15–21)	17 (15–19)	59 (5.6)	8 (3–14)	29 (2.7)	8.79 (7.05 to 10.53)	< 0.001

*Represent the standard categories and the score of the GAD-7; IQR, Interquartile range; CI, Confidence interval

**Table 3:** Change in depression scores based on severity

Depression severity*	Pre-Intervention		Post-Intervention		Absolute Difference (95% CI)	*P* Value
	Median (IQR)	**N** (%)	Median (IQR)	*N* (%)		
None-minimal (Score 0–4)	2 (0–3)	573 (54)	1 (0–3)	757 (71.3)	-0.19 (-0.41 to 0.02)	0.962
Mild (Score 5–9)	7 (6–8)	297 (28)	3 (1–6)	217 (20.5)	2.85 (2.43 to 3.27)	< 0.001
Moderate (Score 10–14)	12 (10–13)	133 (12.5)	6 (3–9)	59 (5.6)	5.28 (4.48 to 6.08)	< 0.001
Moderately Severe (Score 15–19)	17 (16–18)	46 (4.3)	6.5 (2–13.5)	21 (2.0)	8.76 (6.92 to 10.6)	< 0.001
Severe (Score 20–27)	20 (17.3–22.25)	12 (1.1)	10 (2.75–20.25)	7 (0.7)	9.08 (3.64 to 15.18)	< 0.001

*Represent the standard categories and the score of the PHQ-9; IQR, Interquartile range; CI, Confidence interval

**Fig. 3:**
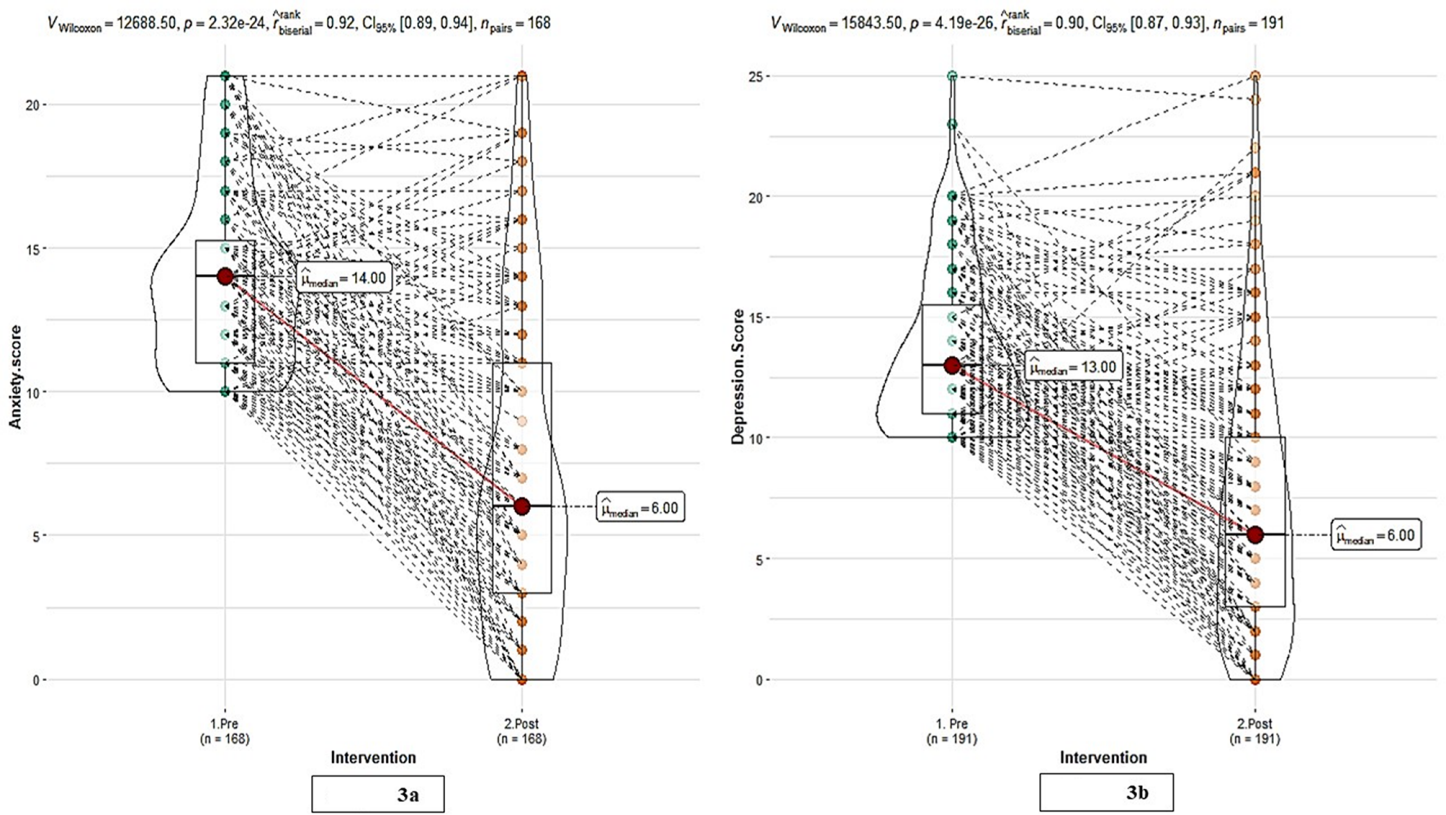
Post-intervention changes in scores in those with moderate to severe conditions at baseline (**a**) anxiety scores (**b**) depression scores

Post-intervention, changes in scores in those with moderate-to-severe symptoms at baseline were analyzed (Fig. 3). Among the 168 patients with moderate-to-severe anxiety at baseline, 69.6% demonstrated an improvement in their scores post-intervention, with the median score decreasing from 14 to 6 (Fig. 3a). Likewise, among the 191 patients with moderate-to-severe depression at baseline, 70.2% exhibited improvement in their scores following the intervention, with the median score dropping from 13 to 6 (Fig. 3b). The effect sizes were d = 1.2 and 1.1 for anxiety and depression, respectively, indicating a substantial impact of the intervention on both conditions. Effect sizes above 0.8 are regarded as large, suggesting a strong and clinically meaningful reduction in anxiety and depression in patients with moderate to severe baseline symptoms.

Individual counseling was provided to those with moderate to severe anxiety and depression. Further analysis to test the efficacy of individual counseling revealed that patients with anxiety at baseline (*n* = 168) showed a significant improvement in their scores, with a median decrease from 14 to 5 with counseling and 14 to 7 without counseling. Similarly, in patients with depression at baseline (*n* = 191), there was a significant improvement in scores; the median score decreased from 13 to 6 for those who received individual counseling and from 12 to 6.5 for those who opted not to receive counseling. The change in median scores was similar in those who received individual counseling versus those who did not (*p* > 0.1).

## Discussion

This retrospective analysis investigated the efficacy of an online integrated ILI for diabetes management to alleviate anxiety and depression in individuals with T2D. The high prevalence of both anxiety and depression symptoms in the study cohort underscores the need for an effective intervention that we addressed through a multidisciplinary approach that showed promising results. The study outcomes revealed marked improvements in the symptoms of anxiety and depression, with the largest reductions observed in individuals experiencing moderate to severe symptoms.

Our study identified a high prevalence of mild to severe depression (46%) and anxiety (44.3%) in our cohort, which was higher than that previously reported for the Indian population [46–49]. This may be attributed to the use of a cutoff score of 5, which includes individuals with mild to severe symptoms of depression and/or anxiety [24, 25], which are often overlooked in studies using higher clinical cut-offs. With a cutoff of 10, the prevalence decreased to 18% for depression and 15.8% for anxiety at baseline which is comparable to other studies [49–51]. The higher prevalence of depression compared to anxiety aligns with previous reports [48, 52]. Post-intervention, participants experienced significant reductions in anxiety and depression scores, with a median drop of two points each. The intervention notably improved severe anxiety, with a substantial decrease of 8.79 points, consistent with the effectiveness of CBT for severe anxiety [40, 53]. For depression, the largest reductions were in the moderately severe to severe groups, with median decreases of 8.76 and 9.08 points, respectively. These findings demonstrate that the intervention was particularly effective in patients with more severe symptoms at baseline, leading to significant reductions in both anxiety and depression scores. Furthermore, the large effect sizes of 1.2 and 1.1 for anxiety and depression highlight the intervention’s substantial impact. These findings align with prior research demonstrating the efficacy of lifestyle modifications, encompassing adjustments to diet and physical activity, in reducing depressive symptoms [54].

Lifestyle interventions, including physical activity, dietary changes, and stress management, improve mental health by improving physiological and psychological well-being [55, 56]. This comprehensive approach addresses the diverse challenges of T2D affecting both physical and psychological health [4]. Previous study has reported significant improvements in anxiety, depression, weight, BMI, and HbA1c levels compared with conventional methods [54]. The efficacy of healthy lifestyle interventions in reducing stress and depression among T2D patients is well-documented [55]. Furthermore, our findings indicate that there was a significant reduction in scores for both anxiety and depression irrespective of individual counseling, suggesting that, although individual counseling contributed to positive outcomes, it may not be the sole factor driving improvement. The comprehensive ILI program, which incorporated components such as exercise, meditation, and yoga, appears to have played a more substantial role in reducing the symptoms of anxiety and depression. These components likely contribute to improved mental health through the reduction of stress hormones, enhancement of mood, and promotion of overall well-being [31, 57, 58]. Improvements in diet and eating habits are also known to enhance blood sugar control and reduce inflammation, contributing to a reduction in anxiety and depression [55]. The ILI also provides social support through group sessions, which can buffer against stress and help people cope with chronic health conditions [59–61]. The group sessions included guided meditation, journaling, and activities promoting positive energy, leading to a calmer mind-body state [62]. A focus on positive emotions through activities such as vision boards contributed to positive outcomes [35, 63]. Further, we chose an online mode for delivery of the intervention because it provides a flexible and accessible means of delivering health-related support with the potential to reach a wide audience at a lower cost. They can also enhance user engagement and self-management capabilities, which are essential for improving health outcomes. However, the implementation of such interventions must consider the associated challenges to maximize their effectiveness [64–68].

The key advantage of this research lies in its lifestyle intervention, significant sample size, and broader geographical coverage compared with earlier Indian studies. However, this study has some limitations, including the lack of a control group and the ability to assess only short-term post-intervention effects. Future prospective studies with longer follow-up periods and control group are necessary to confirm these findings, evaluate the sustainability of the intervention, and explore the mechanisms underlying the outcomes. Despite the large sample size, the retrospective design of our study may have led to a selection bias and confounding factors, which could limit the generalizability of our findings to the broader Indian population. Nevertheless, the online nature of the program allowed us to engage participants from 175 cities across India, potentially expanding the scope of our study and providing a more diverse sample. Additionally, the online program’s subscription model may limit access to those with the financial means to afford it, affecting the generalizability of the results. Furthermore, the success of the intervention depended on patient participation and adherence to lifestyle changes. Despite these limitations, this study provides valuable insights into the prevalence of anxiety and depression among individuals with diabetes in India and the effectiveness of lifestyle interventions in addressing these concerns.

## Conclusion

Online lifestyle-driven multidisciplinary interventions significantly improved the symptoms of anxiety and depression in patients with T2D. Our study contributes to growing evidence that lifestyle modifications may effectively manage these mental health challenges. Future research with a robust design and long-term follow-up is essential to confirm these findings and explore the underlying mechanisms.

## Data Availability

All data supporting our findings have been presented in the manuscript, and the datasets used and/or analyzed during the current study are available from the corresponding author upon reasonable request.

## Abbreviations

T2D: Type 2 diabetes
ILI: Intensive Lifestyle Intervention
PHQ-9: Patient Health Questionnaire
GAD-7: Generalized Anxiety Disorders
CTRI: Clinical Trial Registry of India
ADA: American Diabetes Association
REBT: Rational Emotive Behavior Therapy
NLP: Neuro-Linguistic Programming
CBT: Cognitive Behavior Therapy
SPSS: Statistical Package for the Social Sciences
IQRs: Interquartile Range
OHAs: Oral Hypoglycemic Agents
BMI: Body Mass Index
FBG: Fasting Blood Glucose
PPBG: Postprandial Blood Glucose
